# Diagnostic Value of Fecal Calprotectin (S100 A8/A9) Test in Children with Chronic Abdominal Pain

**DOI:** 10.1155/2016/8089217

**Published:** 2016-11-15

**Authors:** Stanisław Pieczarkowski, Kinga Kowalska-Duplaga, Przemko Kwinta, Przemysław Tomasik, Andrzej Wędrychowicz, Krzysztof Fyderek

**Affiliations:** ^1^Department of Pediatrics, Gastroenterology and Nutrition, Pediatric Institute College of Medicine, Jagiellonian University, Cracow, Poland; ^2^Department of Pediatrics, Pediatric Institute College of Medicine, Jagiellonian University, Cracow, Poland; ^3^Department of Clinical Biochemistry, Pediatric Institute College of Medicine, Jagiellonian University, Cracow, Poland

## Abstract

*Objectives*. The aim of the study was to establish whether fecal calprotectin concentration (FCC) may be useful in children with chronic abdominal pain to differentiate between inflammatory bowel disease (IBD), other inflammatory gastrointestinal disorders, and functional gastrointestinal disorders.* Methods*. The study included 163 patients (median age 13 years), who were assigned to four study groups: group 0 (control), 22 healthy children; group 1, 33 children with functional gastrointestinal disorders; group 2, 71 children with inflammatory gastrointestinal disorders other than IBD; group 3, 37 children with IBD. FCC was measured using ELISA assay.* Results*. In group 0 and group 1 FCCs were below 100 *μ*g/g. Low FCCs were found in 91% of patients in group 2. In patients with IBD FCCs were markedly elevated with median value of 1191.5 *μ*g/g. However, in children with inflammatory gastrointestinal disorders other than IBD and in children with IBD mean FCCs were significantly higher compared with the control group. Significant differences in FCCs were also found between group 1 and group 2, between group 1 and group 3, and between group 2 and group 3.* Conclusion*. FCC is the best parameter allowing for differentiation between IBD, other inflammatory gastrointestinal disorders, and functional gastrointestinal disorders. High FCC is associated with a high probability of IBD and/or other inflammatory gastrointestinal disorders, and it allows excluding functional gastrointestinal disorders.

## 1. Introduction

Chronic abdominal pain in children is a very frequent and sometimes challenging diagnostic issue. Abdominal pain may be constant or intermittent of variable severity and locations. Lack of other features indicative of gastrointestinal disease makes it difficult to establish a final diagnosis. Differential diagnosis of chronic abdominal pain should include not only disorders of the gastrointestinal system (e.g., gastritis and/or duodenitis, pancreatitis, hepatitis, inflammatory bowel disease, celiac disease, food allergy, or gastrointestinal infection) causing inflammatory changes in the gastrointestinal tract, but also diseases of other organs and tissues that are not related to the gastrointestinal system but are located in the abdomen (e.g., urinary tract infection, urolithiasis, and gynecological infections). It is assumed that 14% to 20% of cases of abdominal pain in children are functional [1]. In children over one year of age the most frequent functional gastrointestinal disorders include functional abdominal pain, functional abdominal pain syndrome, irritable bowel syndrome, abdominal migraine, and functional dyspepsia [2].

Patients with functional gastrointestinal disorders present with various signs and symptoms, which are often concerning. However, these manifestations have no organic cause and they require extensive diagnostics based on clinical criteria, results of physical examination, and key diagnostic studies. In practice, diagnostics of the functional disorders is frequently based on exclusion of organic causes, which implies a more or less invasive approach including radiological and endoscopic studies. Majority of patients with chronic and recurrent abdominal pain is unnecessarily exposed to numerous, time-consuming, expensive, and frequently stressful diagnostic studies [3]. Avoidance of procedures that are often associated with various risks is of key importance, particularly in the youngest patients.

There is an ongoing search for simple noninvasive tests that may be used to differentiate between organic and functional gastrointestinal disorders in children. The usefulness of such tests as lactoferrin, eosinophil cationic protein, nitric oxide, or mucosal cytokine levels was evaluated in clinical trials [4–6].

Measurements of fecal calprotectin concentration (FCC) may be useful in the differential diagnosis of various gastrointestinal disorders both in adults and in children [7–15].

Calprotectin is a neutrophil protein that constitutes approximately 60% of neutrophil cytosol proteins. It belongs to the S100 family of proteins and has two subunits: the 8 kD myeloid-related protein 8 (MPR8) and the 14 kD myeloid-related protein 14 (MPR14). It is also found in macrophages, monocytes, and eosinophils [16]. Its properties include binding of calcium and zinc, induction of apoptosis of cancer cells, and inhibition of growth of bacteria and fungi [17].

Neutrophils that are the key cells forming inflammatory infiltrates in the gastrointestinal tract do not actively secrete calprotectin, but they release it in case of cell death or injury [18]. This leads to apoptosis of other cells. The increase in FCC in persons with gastrointestinal inflammation is thought to be due to increased numbers of leukocytes in gastrointestinal mucosa and increased migration of neutrophils to the intestinal lumen.

FCC correlates with the extent of neutrophil infiltrates in intestinal mucosa and with severity of inflammation [7, 19]. Moreover, calprotectin does not undergo enzymatic degradation or modification by drugs used in therapy. In the collected specimens calprotectin levels remain stable for as long as 7 days in ambient temperatures [20].

The usefulness of FCC as a marker of mucosal inflammation has been proven by several studies and meta-analyses [21, 22]. Fecal calprotectin is used in the diagnostics of inflammatory bowel disease (IBD) and particularly of Crohn disease [23, 24], in monitoring of mucosal healing [25, 26], and as a prognostic factor in exacerbations of IBD [27]. Results of clinical studies confirm that, in contrast to inflammatory diseases, FCC remains low in patients with functional gastrointestinal disorders [8, 28–30]. Studies assessing the value of FCC as a screening test in pediatric patients and particularly those with chronic abdominal pain are limited.

## 2. Aim of the Study

The aim of the study was to establish whether in children with chronic abdominal pain FCC may be used to differentiate between IBD, organic inflammatory gastrointestinal disorders other than IBD, and functional gastrointestinal disorders and therefore to reduce the need for burdensome invasive diagnostic studies.

## 3. Patients and Methods

### 3.1. Patients

We conducted a prospective controlled study of 141 children with chronic abdominal pain who fulfilled the following inclusion criteria:

1. Abdominal pain lasting more than 4 weeks with or without other symptoms, including chronic diarrhea, constipation, and weight loss.
2. No prior diagnosis and pharmacological treatment.
3. Age from 4 to 18 years.
4. No overt gastrointestinal bleeding and in girls also no menstrual bleeding within 7 days prior to enrollment.
5. No concomitant features of acute infection (e.g., upper or lower respiratory tract infection).
6. Informed consent of parents/guardians and informed consent of a patient over 16 years.

Exclusion criteria were treatment with antibiotics or with nonsteroidal anti-inflammatory drugs (NSAIDs) in the month preceding the enrollment.

History of current signs and symptoms and prior treatment was recorded on standardized forms that included personal identification data, clinical parameters, and 57 questions concerning symptoms, physiological functions, and school performance and daily activities.

The control group consisted of children who had no abdominal pain within the prior month, received no NSAIDs, and in the case of girls had no menstrual bleeding within prior 7 days.

Patients and their parents/guardians were informed about the aim of the study and provided their written consent to participate. The study was approved by the Ethical Committee of the Jagiellonian University.

### 3.2. Collection of Specimens and the Diagnostic Studies

Fecal specimens were collected to dedicated containers. Within 15 to 30 minutes of collection they were frozen to −20°C and stored until the tests were performed.

Fecal calprotectin was measured with enzyme-linked immunosorbent assay (ELISA) using* PhiCal test* (Calpro AS, Oslo, Norway). The advantages of the assay were its noninvasive character, stability of calprotectin in ambient temperature for up to 7 days, and small volumes of samples necessary to perform the test.

In each patient the following tests were performed: complete blood count (CBC) with differential blood cell count, erythrocyte sedimentation rate (ESR), serum electrolyte concentrations, liver function tests (aspartate aminotransferase, AST, and alanine aminotransferase, ALT), and kidney function tests (urea, creatinine). In most patients the following tests were also performed: C-reactive protein (CRP), endomysial antibodies (EmA), a panel of IBD antibodies: anti-neutrophil cytoplasmic antibodies (pANCA) and anti-*Saccharomyces cerevisiae* antibodies (ASCA), fecal tumor necrosis factor (TNF) alpha, and fecal microbiological, mycological, and parasitological studies, as well as fecal occult blood test and fecal* Giardia lamblia* antigen assay.

In some children other studies were also performed, including abdominal ultrasound, lactulose and/or lactose breath tests, and, when indicated, endoscopic studies with histological examination of biopsy specimens (gastroscopy with urease test and/or colonoscopy).

All the studies were performed at the Department of Clinical Biochemistry, Pediatric Institute College of Medicine Jagiellonian University, Cracow.

### 3.3. Statistical Analysis

Quantitative studies were compared using chi-square test and Fisher exact test. Because of significantly nonnormal distribution of some of the analyzed parameters, variance analysis (ANOVA) was used in the statistical analysis of quantitative variables. Statistical analysis was performed using the IBM SPSS Statistics 21 software. The differences were statistically significant if the risk of the Type I error was below 5% (*α* = 0.05).

## 4. Results

The final analysis included 163 children of a median age of 13 years (25–75 percentile range: 9–16 years). On the basis of their final diagnosis the patients were assigned to one of the 3 following groups:

(1) Children with functional gastrointestinal disorders (*n* = 33).
(2) Children with inflammatory gastrointestinal disorders other than IBD (*n* = 71).
(3) Children with IBD (*n* = 37).

The control group (group 0) included 22 healthy children.

In patients assigned to group 1 the diagnosis of functional gastrointestinal disorders was based on the Rome III criteria [31]. Group 2 included patients with inflammatory gastrointestinal disorders other than IBD, which included esophagitis, gastritis, duodenitis,* Helicobacter pylori* infection, small intestinal bacterial overgrowth (SIBO), pancreatitis, or hepatitis. Group 3 consisted of patients with IBD diagnosed according to the Porto criteria [32].

Characteristics of the study population were as follows: group 0 (control) included 22 children (male/female, 7/15, median age 6.5 years); group 1 included 33 children (male/female, 23/10, median age 12.5 years); group 2 included 71 children (male/female, 51/20, median age 14 years); and group 3 included 37 children (male/female, 18/19, median age 14 years).

**Figure 1:**
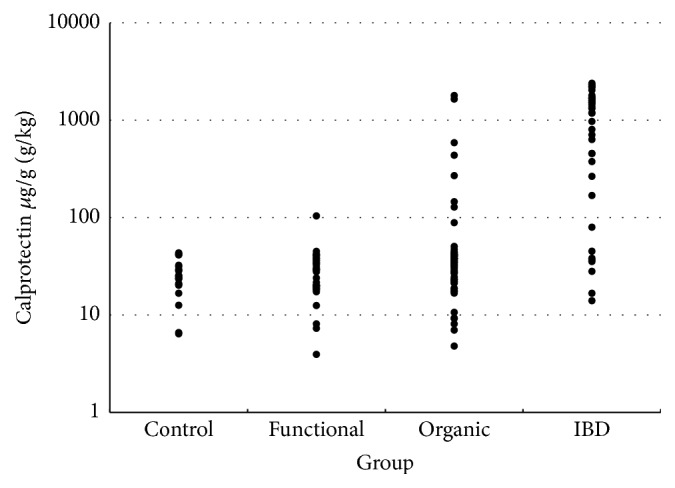
Distribution of fecal calprotectin concentrations (FCC) in the study groups.

In all children in the control group and in all patients with functional gastrointestinal disorders FCC were below 100 *μ*g/g. Moreover, low FCCs were found in 91% of patients in group 2, whereas in most patients with IBD the FCCs were markedly above the cutoff value (median level 1191.5 *μ*g/g; 25–75 percentile range: 265.2 *μ*g/g–1684.9 *μ*g/g) (Figure 1).

No significant differences in FCC were found between the control group and group 1 (*p* = 0.744). However, in children with inflammatory gastrointestinal disorders and IBD mean FCCs were significantly higher compared with the control group (*p* = 0.019 and *p* < 0.001, resp.). Significant differences in FCCs were also found between group 1 and group 2 (*p* = 0.031), between group 1 and group 3 (*p* < 0.001), and between group 2 and group 3 (*p* < 0.001). Although the median values in group 1 and group 2 were similar (22.15 *μ*g/g versus 32.3 *μ*g/g), 25–75 percentile range in group 1 (patients with functional gastrointestinal disorders) was 19,7 *μ*g/g–29.6 *μ*g/g, compared with 23 *μ*g/g–88,7 *μ*g/g in group 2 (patients with inflammatory gastrointestinal disorders). Statistical analysis (chi-square test) revealed significance between group differences (Table 1). Detailed features of group 2 (patients with inflammatory gastrointestinal disorders other than IBD) with FCC distribution are presented in Table 2. FCCs in patients with IBD were significantly higher compared with all other study groups (Table 1 and Figure 1). Group 3 (patients with IBD) included mostly patients with ulcerative colitis (UC; *n* = 17) and patients with Crohn disease (CD; (*n* = 15)) but also 5 patients with unclassified colitis. Median FCC in patients with UC was 1515.0 *μ*g/g, and 25–75 percentile range was 456.1–1724.0 *μ*g/g. Median FCC in patients with CD was 1442.0 *μ*g/g and 25–75 percentile range was 1183.0–1667.2 *μ*g/g. No statistically significant differences between these groups were found. In patients with IBD no correlation between the extent of IBD lesions and FCC was found. In patients with UC no statistically significant differences between patients with left-sided colitis and pancolitis were found. In patients with CD no statistically significant differences between patients with L1, L2, and L3 locations (based on Paris classification) were found. A significant positive correlation between UC Mayo endoscopic score and FCC was seen (Spearman test *r* = 0.47; *p* = 0.025). Patients with new-onset IBD tended to have higher FCC values than patients with chronic disease (median: 1437 *μ*g/g versus 634 *μ*g/g; *p* = 0.07).

Sensitivity and specificity of FCC levels >45 *μ*g/g for the diagnosis of nonfunctional gastrointestinal disorders were 83.8% and 85.9%, respectively (positive predictive value [PPV] 0.75; negative predictive value [NPV] 0.91). Assuming higher FCC (>100 *μ*g/g) as a cutoff point resulted in increased specificity of the test (90%) but it reduced its sensitivity (78%) (PPV 0.80; NPV 0.88).  Figure 3 presents a receiver operating curve (ROC) for the difference between patients with and without IBD.

**Figure 2:**
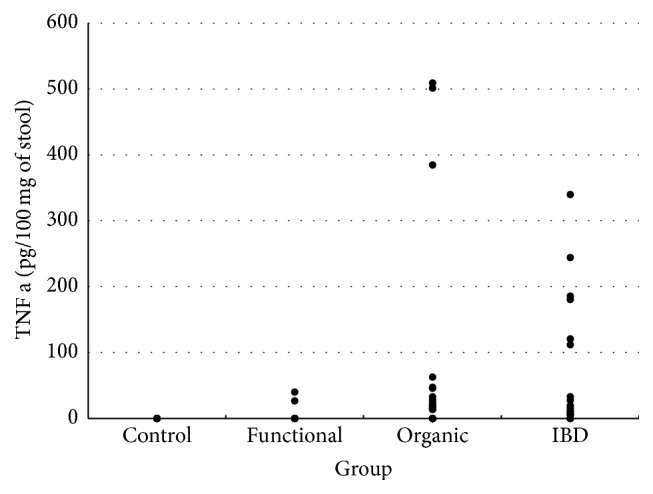
Distribution of fecal tumor necrosis alpha (TNF alpha) levels in the study groups.

**Figure 3:**
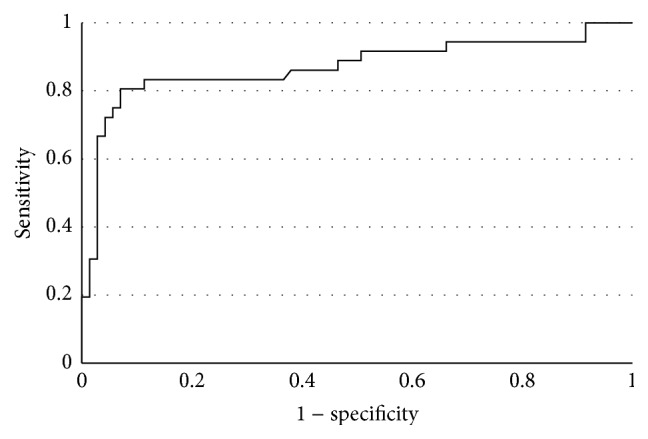
Receiver operating characteristic (ROC) for fecal calprotectin concentrations.

High TNF alpha concentrations were found in group 3 (median: 19.5 pg/mg, Q1–Q3 range: 6.4–120.8 pg/mg) and in group 2 (median: 13.85 pg/mg, Q1–Q3 range: 0–32.9 pg/mg) (Figure 2). These values were significantly higher compared with other groups. CRP levels were measured only in patients undergoing diagnostic workup of abdominal pain and therefore were not assessed in the control group. Comparative levels of calprotectin, TNF alpha, and CRP in the study groups are presented in Table 1.

## 5. Discussion

One of the key diagnostic issues in patients with chronic abdominal pain is to differentiate between functional and organic etiology of the symptoms. Diagnostic workup should focus on a prompt diagnosis of IBD in patients with this disease, as prolonged diagnostics and lack of appropriate treatment increase the risk of complications and lead to delayed growth and puberty in pediatric patients. On the other hand, numerous unnecessary diagnostic studies should be avoided in patients with functional disorders.

In our study the diagnosis of IBD was made in 25% of patients and in approximately half of the participants abdominal pain had organic causes. In 23% of patients abdominal pain was caused by functional gastrointestinal disorders. In these patients and in the control group low FCCs were found.

In the studies published to date various FCC cutoff points were adopted. Most frequently these were <50 *μ*g/g or <100 *μ*g/g. Tibble et al. made a diagnosis of irritable bowel syndrome (IBS) in more than half of the patients consulted by gastrologists for abdominal pain. They adopted the cutoff point at 30 mg/L (150 *μ*g/g), and sensitivity and specificity of FCC as a screening test for organic gastrointestinal disorders were found to be 100% and 97%, respectively [29]. Another study (Carroccio et al.) revealed that the highest diagnostic yield for the diagnosis of CD was achieved when the cutoff point was assumed at 170 *μ*g/g. With this cutoff point sensitivity and specificity of the test were 100% and 95%, respectively. The authors noted that only one of the 15 children eventually diagnosed with IBS had FCC > 50 *μ*g/g [8]. Other studies also revealed that adopting FCC cutoff point of 150–160 *μ*g/g increased specificity of the test with respect to the diagnosis of IBD [30, 33]. A meta-analysis of 13 studies (including 7 pediatric studies) published by Van Rheenen et al. revealed that sensitivity and specificity of FCC for the diagnosis of IBD in pediatric population were lower than in adults and were 93% and 96% versus 92% and 76%, respectively. On the basis of FCC as a screening test, endoscopy would be performed in 65 instead of 100 children, but such an approach would delay the diagnosis in 8% of patients [21]. The authors adopted FCC cutoff point at the level of 50 *μ*g/g. Adopting a similar cutoff value in our study resulted in a slightly lower sensitivity and higher specificity. Similar to other studies we have confirmed that high FCCs (>100 *μ*g/g) suggest exclusion of functional disorders [8]. In our study FCCs > 100 *μ*g/g were not seen in any of the patients in the control group or patients with functional gastrointestinal disorders. Adopting higher cutoff values (>100 *μ*g/g) increases specificity, but not sensitivity of the test. In a small proportion of our patients with IBD (*n* = 7) FCCs were below 100 *μ*g/g. On the other hand, higher FCCs were also found in some of the patients with other inflammatory gastrointestinal disorders. This suggests that FCC is not specific to IBD only and that other inflammatory gastrointestinal disorders (including SIBO, gastrointestinal infections, cow milk protein allergy, or celiac disease) may sometimes cause increased values of this parameter [33–35].

Results of our study demonstrated that FCC has superior value in the differentiation between functional and inflammatory gastrointestinal disorders compared with fecal TNF alpha and serum CRP levels. However, as FCC is not a perfect marker, diagnostic procedures must be selected with caution and decisions to perform further invasive diagnostics should include data from patient's history and other diagnostic studies.

Our study excluded patients with overt gastrointestinal bleeding, as this is one of the “red flag” features and an indication for endoscopy, as well as patients with chronic diarrhea. Pediatric patients with chronic abdominal pain who present no “red flag” features are the most frequent cause of diagnostic dilemmas for both general practitioners and gastroenterologists, as they have equivocal indications of invasive diagnostics. Measurements of FCC facilitate differentiation between IBD, other inflammatory gastrointestinal disorders, and functional gastrointestinal disorders.

In pediatric population and particularly in young children, it is very important to reduce the use of invasive diagnostic procedures, such as gastroscopy or colonoscopy. High FCC may indicate the need for invasive diagnostics, while low values of this parameter suggest absence of pathologic lesions in the gastrointestinal mucosa [22].

A study of adult patients revealed that FCC combined with appropriate indications for endoscopy (according to the recommendations of EPAGE, European Panel on the Appropriateness of Gastrointestinal Endoscopy) increased the chance for successful selection of patients who need invasive diagnostics. Patients with FCC > 50 *μ*g/g had significantly higher incidence of lesions revealed by endoscopy [36].

Unlike commonly used markers of inflammation, such as ESR, CRP, or elevated leukocyte or platelet counts, FCC allows for narrowing the search for inflammatory lesions to the gastrointestinal tract. Therefore it is very useful in differentiation between functional gastrointestinal disorders and inflammatory gastrointestinal disorders [8, 21, 28, 30, 37]. The NPV values approaching 90% make it possible to use FCC as a screening test to exclude organic causes of abdominal pain in children, thus allowing avoiding further invasive diagnostics [38, 39].

## Figures and Tables

**Table 1:** Comparison of fecal calprotectin concentration (FCC), fecal tumor necrosis factor (TNF) alpha and serum C-reactive protein (CRP) levels in the study groups.

	Group 0	Group 1	Group 2	Group 3
FCC (*µ*g/g)^*∗*^; median (25–75 percentile range)	25.1 (20.7–31.5)	22.15 (19.7–29.6)	32.3 (23–88.7)	1442 (708–1724)
Fecal TNF alpha^**∗∗**^ **** (pg/100 mg of stool); median (25–75 percentile range)		0.00 (0.00–0.1)	13.85 (0.00–32.9)	19.5 (6.40–120.8)
Serum CRP^**∗∗∗**^ (mg/L); median (25–75 percentile range)		3.00 (2.97–3.00)	3.00 (3.00–3.19)	3.19 (3.05–9.20)

^*∗*^Chi-square test for the variable FCC, *p* < 0.0001.

^*∗∗*^Chi-square test for the variable TNF alpha, *p* = 0.0006.

^*∗∗∗*^Chi-square test for the variable CRP, *p* = 0.0042.

**Table 2:** Detailed characterization of Group 2 (patients with inflammatory gastrointestinal disorders other than IBD) with FCC distributions (*n* = 71).

Group 2	Median FCC (*μ*g/g)	25 percentile FCC (*μ*g/g)	75 percentile FCC (*μ*g/g)
Esophagitis/duodenitis/gastritis	36.6	22.0	42,3
Helicobacter pylori gastritis	37.5	28.6	42,3
SIBO	32.8	27.2	40.2
Gastrointestinal infections, for example, EPEC	39.5	39.0	40.4
Other: hepatitis, pancreatitis	33.2	23.0	41.9

FCC distribution is the same for all subgroups (Kruskal-Wallis test for independent groups).

## Additional Points

*What Is Known*. In adults fecal calprotectin concentration is a useful marker of inflammatory bowel disease and is used to monitor activity of the disease.* What Is New*. (i) In pediatric patients fecal calprotectin concentrations over 50 *μ*g/g suggest exclusion of functional gastrointestinal disorders. (ii) The value of fecal calprotectin as a marker used for differentiation between functional and inflammatory gastrointestinal disorders is superior to fecal tumor necrosis factor alpha and serum C-reactive protein.
